# Characteristics of BeiDou Navigation Satellite System (BDS) Code Observations for Different Receiver Types and Their Influence on Wide-Lane Ambiguity Resolution

**DOI:** 10.3390/s18103546

**Published:** 2018-10-19

**Authors:** Yangwei Lu, Zhenjie Wang, Shengyue Ji, Wu Chen, Duojie Weng

**Affiliations:** 1 Department of Surveying and Mapping, China University of Petroleum (East China), Qingdao 266580, China; lugnss@gmail.com (Y.L.); wzj680209@sina.com (Z.W.); 2 Laboratory for Marine Mineral Resources, Qingdao National Laboratory for Marine Science and Technology, Qingdao 266071, China; 3 Department of Land Surveying and Geo-informatics, The Hong Kong Polytechnic University, Hong Kong, 999077, China; wengduojie@aliyun.com; 4 The Hong Kong Polytechnic University Shenzhen Research Institute, Shenzhen 518057, China; wu.chen@polyu.edu.hk

**Keywords:** BDS, GEO, multipath, Melbourne-Wübbena (MW) combination, wide-lane, ambiguity resolution, different receiver types

## Abstract

The Chinese BeiDou Navigation Satellite System (BDS) has been an important constitute of the Global Navigation Satellite System (GNSS), and the combination of GPS and BDS shows significant improvements when compared with single GPS system for real-time kinematic (RTK) positioning, and improves on availability and fixing rates, especially in the East Asian area. While network RTK might have different types of receivers, both for global and regional networks, different types of receiver may adopt different internal multipath mitigation methods and other techniques that result in different pseudorange characteristics, especially for a multipath. Then, the performance of wide-lane ambiguity resolution (WL AR) is affected. In this study, we first analyze and compare the characteristics of BDS dual-frequency observations for different types of receivers, including Trimble, Leica, Javad, and Septentrio, based on multipath (MP) observables, and then we assess their influence on double-differenced (DD) WL AR. The numerical results show that an obvious low-frequency component exists in MP observables of BDS geostationary earth-orbit satellites (GEOs) for Leica receivers, while its high-frequency measurement noise is very small. For geosynchronous orbit satellites (IGSOs) and medium earth-orbit satellites (MEOs), a slight fluctuation can also be observed that is similar to that of GPS satellites, except for the satellite-included code bias. In Trimble, Javad, and Septentrio receivers, the MP series are dominated by high-frequency measurement noise, both for GEOs and non-GEOs, except for satellite-included code bias. Furthermore, the characteristic of Leica receivers for BDS GEOs seriously affects WL AR and, even for a short baseline, it takes a long time for WL ambiguities to converge, or not converge for many GEO-related DD WL ambiguities, while Trimble, Javad, and Septentrio receivers perform well for short and medium baselines. Then, a time-difference method is proposed to mitigate the multipath of BDS GEOs for a Leica receiver. After applying the proposed method, WL ambiguity fixing rates of GEO-related satellite pairs are improved significantly and the convergence time is shortened from several hours to ten minutes.

## 1. Introduction

The Chinese BeiDou Navigation Satellite System (BDS) has been an important constitute of the Global Navigation Satellite System (GNSS), together with the United States (U.S.) system GPS, the Russian system GLONASS, and the European system Galileo. It officially started to provide continuous positioning, navigation, and timing services for China and the surrounding area on 27 December 2012. Multiconstellation combination brings multiple visible satellites as compared with single system, which provides more redundant observation information [1].

In the Asia-Pacific area, five geostationary earth-orbit satellites (GEOs) of BDS can be observed all the time, together with the six geosynchronous orbit satellites (IGSOs) and three medium earth-orbit satellites (MEOs), which increase the visible satellites. This can significantly shorten first-fix time compared with single GPS system, both for real-time kinematic (RTK) positioning and precise point positioning (PPP), and also improve availability and fixing rates [2,3,4].

As we know, the most widely used commercial positioning technology for real-time high-accuracy positioning is the network RTK, and one of its most basic components is the reference network. Many permanent and continuous GNSS networks, including the International GNSS Service network (IGS), EUREF Permanent Network (EPN), GPS Earth Observation Network (GEONET), and a few other regional GNSS nets can be found all over the world. In Hong Kong, the Continuously Operating Reference Station (CORS) network named ‘SatRef’, established by the Survey and Mapping Office (SMO) of the Land Department of Hong Kong, is also a mixed-receiver network. It usually employs different types of receivers, both for global and regional networks, especially for large networks.

However, different types of receiver may adopt different internal multipath mitigation methods and other techniques, which might result in different measurement performances and precise positioning. Li et al. [5] assessed the stochastic model of different types of receiver of GPS observations, which suggested that the stochastic model is much associated with receiver types and should be specified for those receiver types. Hauschild et al. [6] analyzed the multipath characteristics of the GPS SVN49 satellite with different types of receiver and each of them adopted different multipath mitigation methods, including ‘mpnew’ (Javad Delta TRE-G3TH), Pulse Aperture Correlator (PAC, NovAtel OEMV), Hatch-correlator (NavCom SF3050), and A Posteriori Multipath Estimator (APME, Septentrio PolaRx2). They concluded that the NovAtel OEMV receiver employing the PAC method showed the smallest mitigation effect. The maximum multipath at 90° elevation still amounts to 1.5 m for the L1 C/A-code.

For the network RTK, the model called ‘WL-NL’ is often used to resolve double-differenced ambiguity for reference stations for dual-frequency users; wide-lane ambiguity resolution (WL AR) is the key step of precise relative positioning [7,8,9]. The WL is usually calculated with the Melbourne-Wübbena (MW) combination that is dominated by the multipath and pseudorange measurement noise. The measurement noise can be handled very well with a filter, while the multipath is difficult to mitigate by differencing, modeling, or smoothing.

Furthermore, the BDS is a mixed-constellation navigation satellite system, which results in different measurement performances from different satellite types. Investigations for the signals from MEO satellites began immediately after the launch of the COMPASS-M1 experimental satellite in 2007; MEO satellites share many common features with GPS and Galileo. The satellite’s PRN code sequences were decoded using directive antennas [10]. Test results showed that the observed signal strength of the experimental satellite was stronger than that of the GPS signals [10]. Characterization of Compass M-1 signals was examined by Hauschild et al. [11] with multipath (MP) combination observables that were formed by a single-frequency code measurement and dual-frequency phase measurement. Test results showed that a systematic error of unknown origin was present in the measurements. Since China completed a basic COMPASS navigation network with three GEOs and three IGSOs in orbit in April 2011, signals for three different kinds of satellites were studied. MP analysis, the same one as Hauschild, showed that the noise level of COMPASS GEO and IGSO code measurements were higher than those of GPS satellites as a whole [12].

Moreover, as demonstrated by Wanninger and Beer [13], these satellite-included code bias variations can exceed 1 m; two elevation-dependent models were proposed for IGSO and MEO satellites, respectively. Based on this model, an improved model that takes the stochastic model of corrections into consideration was proposed by Guo, et al. [14]. Lou et al. [15] proposed a third-order polynomial model for GEOs, IGSOs, and MEOs, respectively, to improve the fixing rate of extra-wide-lane (EWL) and WL AR for a long-distance baseline, which is also an elevation-dependent model and it significantly improved the success rate of WL AR. In addition, the effect of BDS code-bias fluctuation on WL AR has been analyzed [16,17,18,19], which indicates that systematic errors may seriously affect the WL AR, especially for GEO satellites. As mentioned by Wanninger and Beer [13], similar code-carrier divergences were found for GPS SVN49, and the level of these code errors depends on the receiver’s multipath mitigation technique [6].

Our objectives in this paper are to characterize the BDS pseudorange measurements for different receiver types and analyze their effect on WL AR. This article is organized, as follows: Section 2 describes the data and methodology in details. Section 3 demonstrates the characteristics of BDS pseudorange observations of Trimble, Leica, Javad, and Septentrio receivers with MP series based on wavelet and correlation analysis. Then, the influence on double-differenced (DD) WL AR is examined. Furthermore, the performance of an improved AL AR algorithm is also shown in Section 3. Finally, discussion and conclusion are given in Section 4.

## 2. Methodology and Datasets

In this section, we give a brief introduction on MP observables and the WL AR of dual-frequency observables because only dual-frequency observations can be obtained for most users. Then, an improved WL AR method to mitigate the low-frequency multipath is introduced.

### 2.1. Code Multipath Observable

The multipath observable is employed to analyze the characteristics of multipath effects, which can be expressed, as follows [20]:

(1) $$MP_{j}=P_{j}-\frac{f_{j}^{2}+f_{i}^{2}}{f_{j}^{2}-f_{i}^{2}}L_{j}+\frac{2f_{i}^{2}}{f_{j}^{2}-f_{i}^{2}}L_{i}-B$$

where subscripts *i*, *j* are the carrier frequencies, $P_{j}$ is the observed pseudorange, $L_{j}$ is the observed phase in meters, *f* denotes the carrier-phase frequency in Hertz, and *B* is the sum of phase ambiguity and the constant part of hardware delay and multipath. 

The multipath combination is both ionosphere-free and geometry-free. After removing the constant term, the residual series mainly contain code multipath and noise, as the systematic errors and noise of the carrier-phase measurements are negligible when compared with those of the code observations.

### 2.2. Wide-Lane Ambiguity Resolution

For dual-frequency users, the Melbourne–Wübbena (MW) combination is usually used to resolve wide-lane ambiguity. The MW combination is a linear combination of both carrier phase and code observables, which is defined as [7,9,21,22,23]:

(2) $$L_{MW}=\frac{1}{f_{1}-f_{2}}\left(f_{1}L_{1}-f_{2}L_{2}\right)-\frac{1}{f_{1}+f_{2}}(f_{1}P_{1}+f_{2}P_{2})$$

The MW combination is both ionosphere-free and geometry-free, which can eliminate the effect of the ionosphere, the geometry, the clocks, and the troposphere, and only measurement noise and multipath remain. 

For double-differenced observations, the double-differenced MW observable can be obtained with:

(3) $$L_{MW,kl}^{ij}\left(n\right)=\lambda_{WL}n_{WL,kl}^{ij}+\varepsilon$$

where $L_{MW,kl}^{ij}$ is a DD MW observable, $\lambda_{WL}$ is the wavelength of the wide lane, $n_{WL,kl}^{ij}$ is wide-lane ambiguity of double-differenced observations, and ε is the sum of noise, multipath, and high-order ionosphere delay.

Usually, the MW combination observable is smoothed with a Hatch filter, which can be expressed, as follows [24]:

(4) $${\bar{L}}_{MW,kl}^{ij}\left(n\right)=L_{MW,kl}^{ij}\left(n\right)/n+{\bar{L}}_{MW,kl}^{ij}\left(n-1\right)*\left(n-1\right)/n$$

where *n* is the continuous epoch number from last cycle slip or gap.

For DD MW combinations, the main errors are measurement noise and the multipath of code and phase observations. Under ideal conditions, we assume that the observation noise and multipath have white-noise characteristics, so MW combinations also have white-noise characteristics. However, it is difficult to have these ideal conditions all the time, for example, the serious multipath will make the MW combination contain a low-frequency component, which will seriously affect WL AR. This is because, if the dominant component of ε is high-frequency noise, wide-lane ambiguity can be resolved directly after smoothing [25]; if the dominant component of ε is a low-frequency component, then wide-lane ambiguity is difficult to be resolved, as it will need a long time to converge or it will not be able to converge.

### 2.3. Improved WL AR Method to Mitigate Low-Frequency Multipath

The commonly used method to mitigate a multipath is the sidereal filter, which employs a wavelet to retrieve the low-frequency component and then substitute it from the next sidereal day. 

If the dominant component is a low-frequency part and the random measurement noise is very small, we use the raw MP value to mitigate the multipath. Considering the measurement noise, the time-difference method was employed to correct the raw pseudorange measurement, that is, the current pseudorange can be corrected with the MP value of a previous epoch.

In order to obtain the MP value, constant bias B can be calculated, as follows:

(5) $$B{\left(k\right)}_{j}=B{\left(k-1\right)}_{j}+\frac{MP{\left(k\right)}_{j}-B{\left(k-1\right)}_{j}}{k}$$

where k is the continuous epoch number from last cycle slip or gap.

Cycle slips were detected with the epoch-difference method based on the MP and MW series. If no cycle slip occurs between two epochs, unknown ambiguity stays the same and the bias is calculated, as in Equation (5). If a cycle slip occurs or the tracking status of a satellite is switched at a certain epoch, then a series of equations as in Equation (5) can be formed into a new block.

The MP value $\tilde{MP}{\left(k\right)}_{j}$ of the k-th epoch is read as:

(6) $$\tilde{MP}{\left(k\right)}_{j}=MP{\left(k\right)}_{j}-B{\left(k\right)}_{j}$$

Then, the raw pseudorange measurements can be corrected with the $\tilde{MP}{\left(k\right)}_{j}$:

(7) $$\tilde{P}{\left(k+1\right)}_{j}=P{\left(k+1\right)}_{j}-\tilde{MP}{\left(k\right)}_{j}$$

We correct the pseudorange of the current epoch with $\tilde{MP}{\left(k\right)}_{j}$ of last epoch other than current epoch directly, because it contains measurement noise, even though the MP series is dominated by a low-frequency multipath.

### 2.4. Datasets

**Figure 1:**
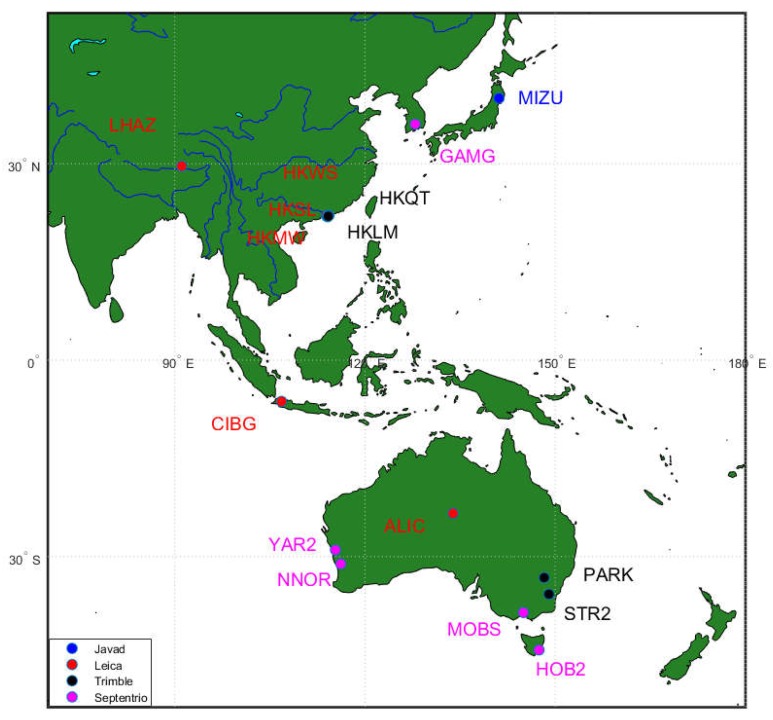
Distribution of stations used in this study.

The observation data used in this study were collected from 16 stations from four different receiver types, as shown in Figure 1; one-week data were collected in every season in 2017 (DOY:072–078, 109–115, 202–208, 294–300).

All stations were selected in the Asia-Pacific region considering the GEOs and baseline distance. There was only one station that MIZU deployed with a Javad receiver, as we did not find other proper stations where GEOs were in view and the baseline distance was short or medium. The other stations were deployed with Leica, Trimble, and Septentrio receivers, respectively. Table 1 shows the detailed information of all stations.

## 3. Experimental Analysis

In this section, we first analyze the characteristics of BDS observations that were based on MP observables for different receiver types, including Trimble, Leica, Javad, and Septentrio. Then, the wavelet and correlation analysis method were adopted to confirm the low-frequency component of GEOs’ MP series for a Leica receiver. After analyzing the influence on WL AR, an improved WL AR method was validated based on a short baseline.

### 3.1. Variations of MP of BDS for Different Receiver Types

**Figure 2:**
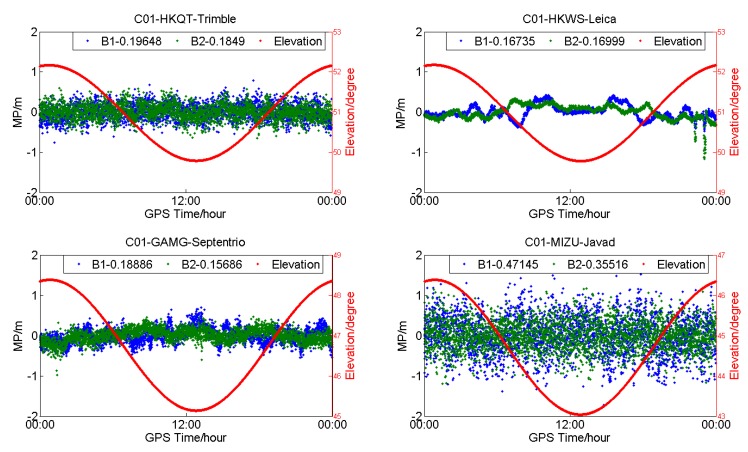
Multipath (MP) (**blue** and **green**) and satellite elevation (**red**) of C01 (geostationary earth-orbit satellites (GEO)) for different receiver types (DOY:203).

**Figure 3:**
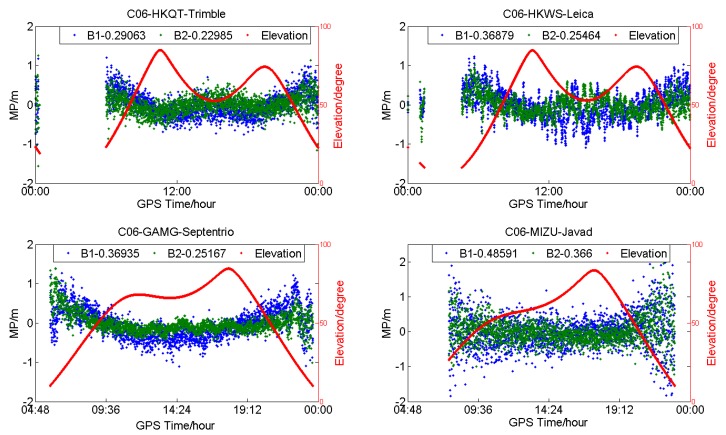
Multipath (**blue** and **green**) and satellite elevation (**red**) of C06 (geosynchronous orbit satellites (IGSO)) for different receiver types (DOY:203).

**Figure 4:**
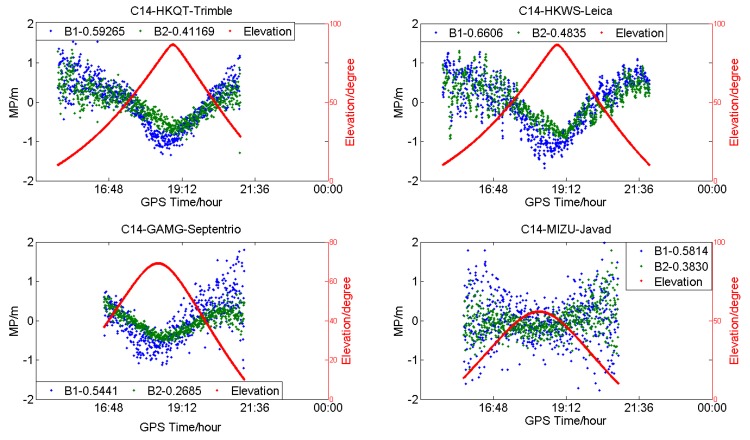
Multipath (**blue** and **green**) and satellite elevation (**red**) of C14 (medium earth-orbit satellites (MEO)) for different receiver types (DOY:203).

Figure 2, Figure 3 and Figure 4 show the MP series of BDS satellites for four different receiver types. As shown in Figure 1, the receiver types of HKQT, HKWS, GAMG, MIZU are Trimble, Leica, Septentrio, and Javad, respectively. Table 1 indicates the details of those four stations, including receiver and antenna types. 

The plots in Figure 2 illustrate the MP series of C01 (GEO) for different stations (DOY:203). It can be observed that the MP series of C01 at HKWS (Leica receiver) is obviously different from others, both for B1 and B2, as its dominant component is a low-frequency multipath and the measurement noise is very small, while for HKQT, GAMG, and MIZU, the dominant component is high-frequency measurement noise. As a result, the standard deviation (STD) of the MP of C01 for HKWS was smaller than that of other stations, as shown in Figure 2. The STD of the MP of B1 for the four stations (HKQT, HKWS, GAMG, MIZU) was 0.19648 m, 0.16736 m, 0.18886 m, and 0.47146 m, respectively. The low-frequency multipath also did not vary with elevation. In addition, the MP series for HKQT (Trimble) and GAMG (SEPT POLARX4TR) had a low-frequency component to some degree, but their frequencies were much higher than that of HKWS, while for MIZU (JAVAD TRE_G3TH), there was no obvious low-frequency component in the MP series, but its measurement noise was much bigger than that of the other stations.

Figure 3 shows the MP series of C06 (IGSO) for HKQT, HKWS, GAMG, and MIZU. Except for the code bias varying with elevation, as described [13,14,15,16], slight fluctuation could also be found of IGSOs for HKWS, which was not the case for HKQT, GAMG, and MIZU. We did not find a similar fluctuation in the other three stations, and it was the same for MEOs, as shown in Figure 4.

**Figure 5:**
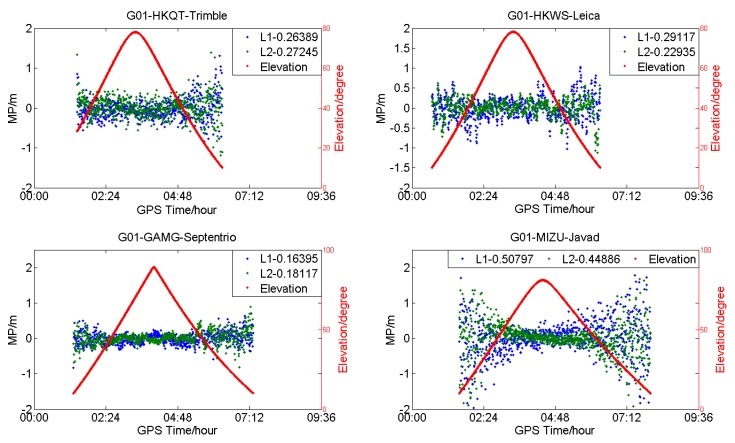
Multipath (**blue** and **green**) and satellite elevation (**red**) of G01 for different receiver types (DOY:203).

To compare with GPS, the MP series of G01 for HKQT, HKWS, GAMG, and MIZU are also shown in Figure 5. A slight fluctuation can also be found for HKWS, which is similar to the MEOs of BDS. The MP series vary smoothing with the elevation for HKQT, GAMG, and MIZU, while it is not the same for HKWS, it seems that it does not follow a normal distribution with zero mean.

**Figure 6:**
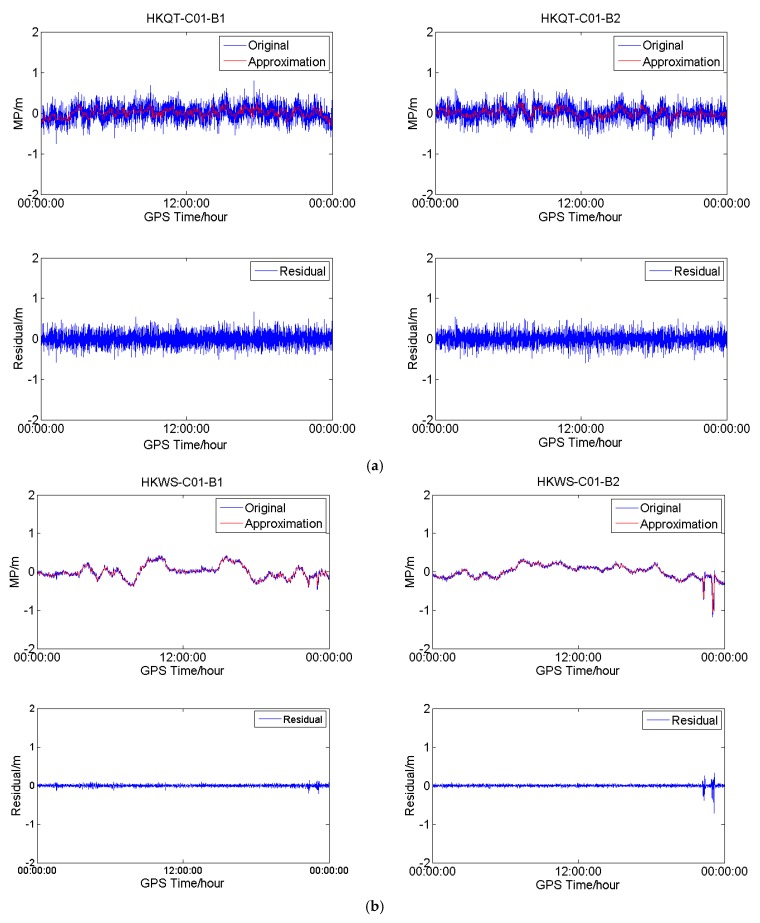

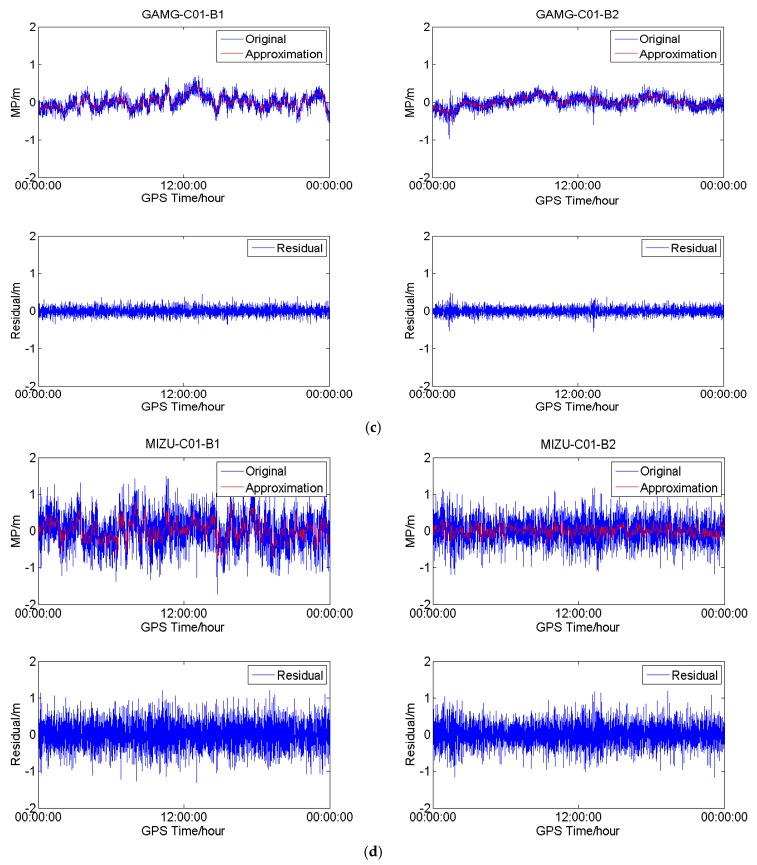
(**a**) Wavelet analysis results for C01 at HKQT (Trimble). (**b**) Wavelet-analysis results for C01 at HKWS (Leica). (**c**) Wavelet-analysis results for C01 at GAMG (Septentrio); and, (**d**) Wavelet-analysis results for C01 at MIZU (Javad). ‘Original’ represents the original multipath series, ‘Approximation’ the approximate or low-frequency components, and ‘Residual’ the difference between the two.

In order to further confirm the existence of the low-frequency component of the MP series of C01 for HKWS, the MP series of C01 for the four stations were processed with wavelet decomposition and reconstruction, and low-frequency components, also called approximate components, were obtained, which are shown in Figure 6. The plots in Figure 6a demonstrate the result of C01 for HKQT (Trimble). The original MP series fluctuated within 0.5 m and obviously contained a low-frequency component, while after the low-frequency parts were removed, the residuals still fluctuated within 0.5 m, which means that the residuals were dominant, that is, the random measurement noise. Results are much different for HKWS (Leica) in Figure 6b. The original MP series and ‘Approximation’ series almost completely overlap, with the residuals being much smaller than the ‘Original’ series. For the GAMG (Septentrio) (Figure 6c) station, the raw MP series also included low-frequency parts, the residuals varied within about 0.35 m, while the ‘Original’ fluctuated within about 0.5 m. Furthermore, the frequency of the low-frequency components was much higher than that of HKWS. Figure 6d shows the results of MIZU (Javad). It can be observed that, after the low-frequency parts were removed, residuals fluctuated a lot as they were random noise, which means that high-frequency measurement noise was the dominant component for MIZU, which is totally different from HKWS (Figure 6b). As a result, we conclude that, for HKWS with a Leica receiver, the low-frequency multipath was the dominant component of MP observables for C01, while for other stations, high-frequency measurement noise was the dominant component.

To characterize the observations of different receivers, all GNSS observations of those four stations have been analyzed, and we found that the observation characteristics for HKWS with a Leica receiver were obviously different from others, especially for GEOs of BDS. There was on obvious low-frequency component in the MP series of GEOs of BDS for HKWS with Lecia receiver.

For non-GEOs of the BDS and GPS satellites of HKWS, the MP series were also slightly different from the other three stations. Relative low-frequency fluctuations could also be observed, but they had almost no difference for the non-GEOs of BDS and GPS satellites, and their frequencies were much higher than those of GEOs. 

As we know, the multipath mainly originates from the environment, satellites, and receiver. If the low-frequency multipath originated from satellites, then this phenomenon would be observed at all stations, which is not true according to the above analysis.

If it originates from the signal refraction of the environment, the receiver deployed in other places may perform well, or at least most of the other stations with a Lecia receiver would perform well. Of course, we must assure that it is indeed the multipath and not an abnormal phenomenon just for one day.

### 3.2. Daily Repeatability and Spatial Similarity

In order to confirm whether the low-frequency variations of the BDS MP series of a Leica receiver originated from a receiver multipath or not, the daily repeatability and spatial similarity of MP observables of GEOs and IGSOs were analyzed for MEOs, whose variations of MP series were very similar to those of GPS, so their daily repeatability and spatial similarity are not shown in this section.

**Figure 7:**
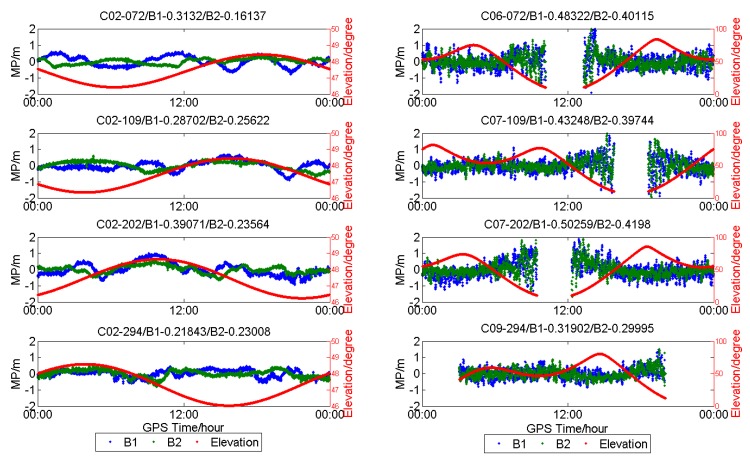
Multipath (**blue** and **green**) and satellite elevation (**red**) of GEOs and IGSOs of HKMW (Leica) station on different days in 2017.

Figure 7 shows the MP series of C02, C06, C07, and C09 of HKMW on one day in each season in 2017. The low-frequency component of the MP series for C02 can be obviously observed during the four days, and the MP series of the three IGSOs also demonstrate a slight fluctuation. 

**Figure 8:**
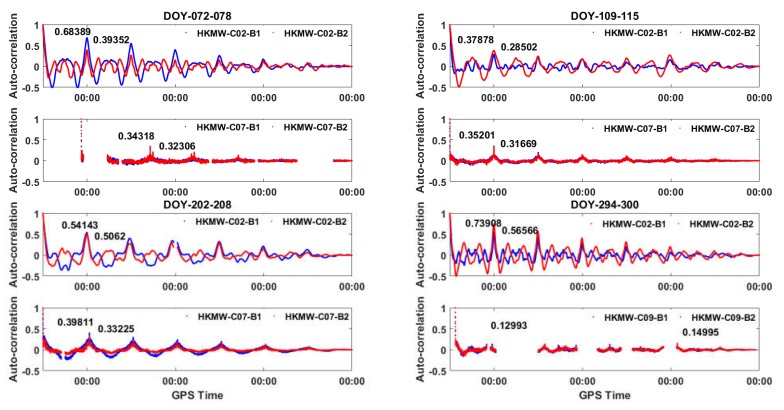
Autocorrelations of seven-day period MP series for GEOs and IGSOs at station HKMW in 2017.

Autocorrelation was performed on the seven-day period MP series of C02, C07, and C09 of HKMW in each season. The normalized autocorrelation values for the multipath series are shown in Figure 8. The peaks reoccurred every day or every half day in every season, suggesting high similarity between waveforms of MP series for two consecutive days, which also indicates that the low-frequency component of an MP series was not an abnormal phenomenon just for one day, but it was indeed the multipath. We also found that the normalized autocorrelation values varied with the season, which is another question and not the emphasis in this paper. 

**Figure 9:**
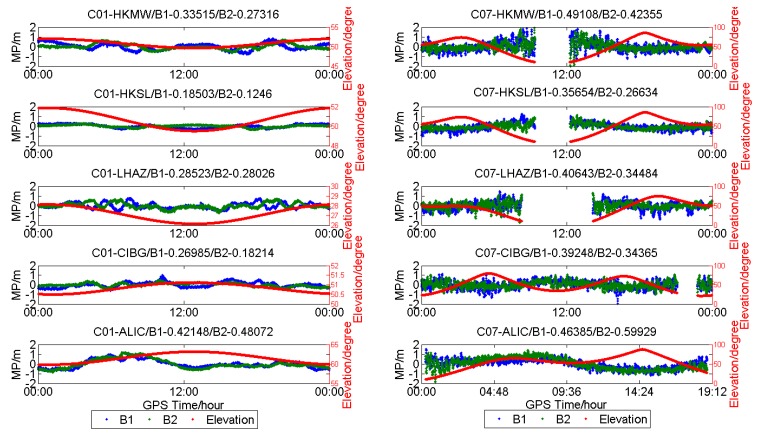
Multipath (**blue** and **green**) and satellite elevation (**red**) of C01 and C07 of different stations (DOY 203).

Figure 9 shows the MP series of C01 and C07 on five stations, HKMW, HKSL, LHAZ, CIBG, and ALIC, as examples. Even though the five stations were deployed in different places, which might have affected the MP series, the low-frequency component of the MP series could also be obviously observed for C01, which did not vary with elevation angle.

**Figure 10:**
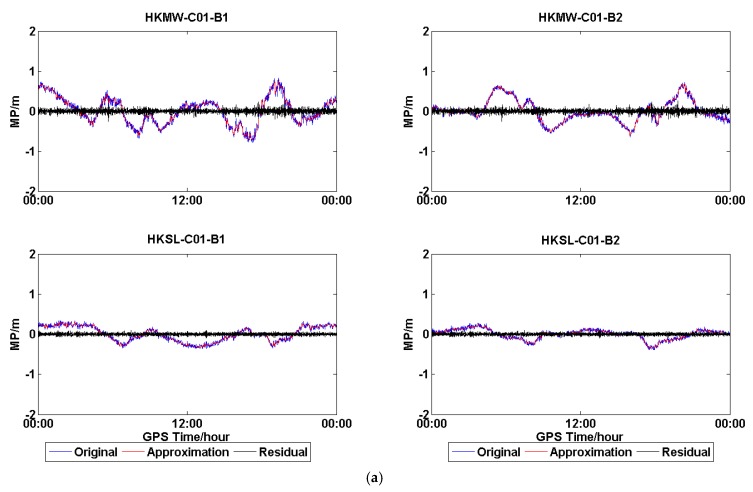

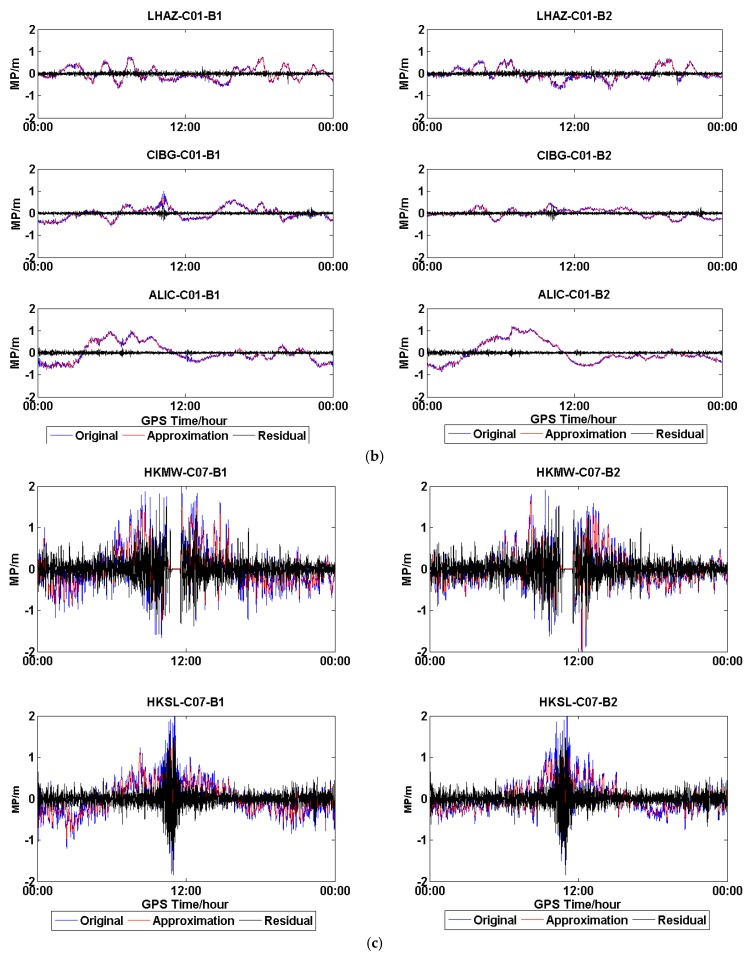

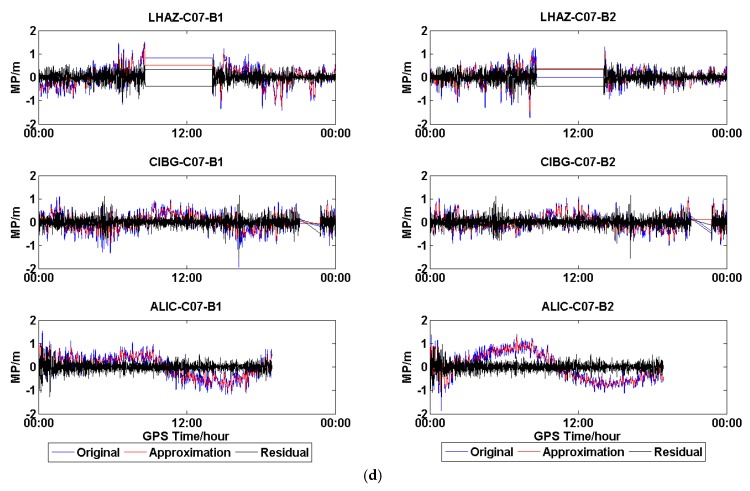
(**a**) Wavelet-analysis results for C01 at HKMW, HKSL. (**b**) Wavelet-analysis results for C01 at LHAZ, CIBG, ALIC. (**c**) Wavelet-analysis results for C07 at HKMW, HKSL. (**d**) Wavelet-analysis results for C07 at LHAZ, CIBG, and ALIC. ‘Original’ represents the original multipath series, ‘Approximation’ the approximate or low-frequency components, and ‘Residual’ the difference between the two.

Wavelet-analysis results are demonstrated in Figure 10. The plots in Figure 10a show the wavelet analysis of C01 of HKMW and HKSL, both for B1 and B2. It is obvious that the low-frequency parts are the dominant components, and the residuals are very small after removing the low-frequency components. Although both HKMW and HKSL are located in Hong Kong, the low-frequency multipath was also different in a different environment. Figure 10b illustrates the low-frequency components and the random measurement noise of the C01 MP series of LHAZ, CIBG, and ALIC, which are located in China, Indonesia, and Australia, respectively. The result is similar to Figure 10a, and a low-frequency multipath can be obviously observed for the three stations, although the stations are located in different countries, it just resulted in different variations of the low-frequency parts. The wavelet analysis results of C07 (IGSOs) for the five stations are demonstrated in Figure 10c,d. Low-frequency multipaths are also observed, though they are less obvious than GEOs. Furthermore, the low frequency of IGSOs might mainly result from satellite-included multipaths. For example, in Figure 10c, the low-frequency parts of HKMW and HKSL almost varied with the same trend, because the satellite-included multipaths varied with elevation, and their elevation angle was almost the same for both located in Hong Kong. 

Overall, Figure 10 suggests that an obvious low-frequency component exists in the MP series of C01 for all five stations with a Leica receiver. A low-frequency component was also observed in the MP series of C07, but it was different from that of C01, as it varied with elevation. A similar phenomenon could also be observed for other GEOs and IGSOs of BDS. 

When considering the above analysis, we can conclude that obvious low-frequency components indeed exist in the MP series of GEOs of BDS for those stations with a Leica receiver. This demonstrates high similarity between two consecutive days, and for the receivers located in different place, a low-frequency component could also be observed.

We have confirmed the above phenomenon with the other stations that deployed Leica receivers, and similar results were obtained, but not for Trimble, Septentrio, and Javad. We argue that this low-frequency multipath might have originated from the receiver, which may result from internal multipath mitigation methods.

### 3.3. Characteristics of BDS MW Combinations for Different Receiver Types

In this section, the fixing rate and the convergence time (time-to-first-fix and reconvergence time) of DD WL AR are assessed with short and medium baselines for different receiver types, except Javad, as there were no proper data available. Then, a time-difference multipath-mitigation method for GEOs of a Leica receiver was proposed. DD MW combinations only contained measurement noise and multipath, so it could also be used to characterize the measurements of different receiver types.

In order to analyze the effect of a multipath on WL AR, six baseline datasets (DOY: 072–078, 109–115, 202–208, 294–300) that contained 11 stations belonging to three receiver types, Trimble, Leica, and Septentrio, were collected. For the Javad receiver, we did not find proper short baseline data with GEOs. The distances of those baselines are shown in Table 2 and all of the stations are shown in Figure 1.

**Figure 11:**
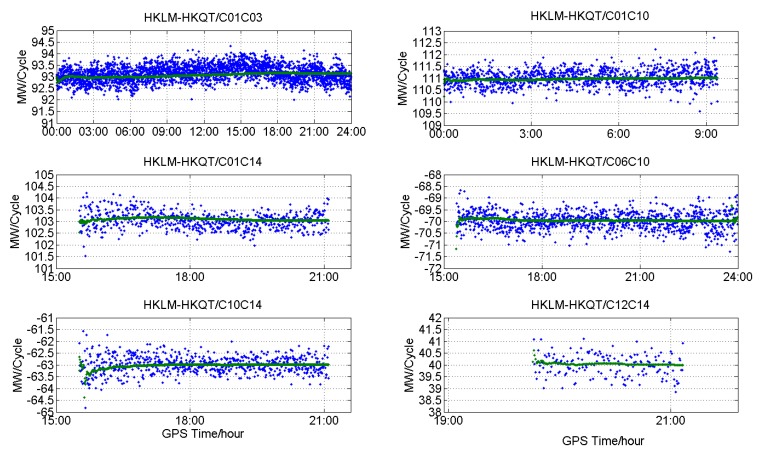
Double-differenced Melbourne–Wübbena (DD MW) combinations for different types of satellites of baseline HKLM-HKQT (Trimble).

**Figure 12:**
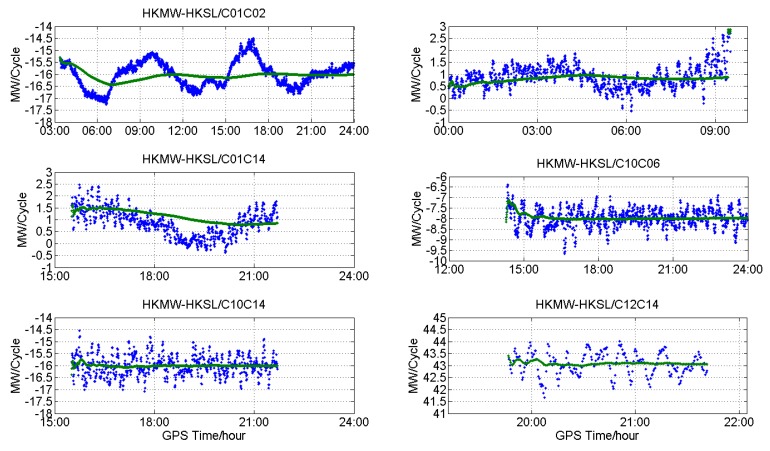
DD MW combinations for different types of satellites of baseline HKMW-HKSL (Leica).

**Figure 13:**
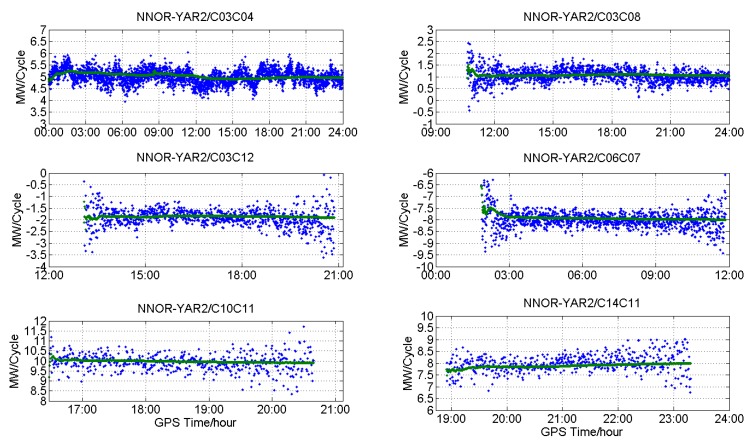
DD MW combinations for different types of satellites of baseline NNOR-YAR2 (Septentrio).

Figure 11, Figure 12 and Figure 13 show the DD MW combinations (in cycles) of three baselines, HKLM-HKQT (Trimble), HKMW-HKSL (Leica), NNOR-YAR2 and (Septentrio), the green lines are float wide-lane ambiguities after smoothing with a Hatch filter [24], and the blue dots are single-epoch MW combination observables. For the Septentrio receiver, we did not find short baseline data, so the MW combination observables of a medium baseline are shown in Figure 13.

As shown in Figure 11, Figure 12 and Figure 13, MW combinations of baseline HKWS-HKSL (Leica) are obviously different from baseline HKLM-HKQT and NNOR-YAR2. The MW combination series of baseline HKMW-HKSL (Leica) in Figure 12 indicates that obvious fluctuations of low-frequency multipath components can be found, while the measurement noise was very small compared to the other two baselines, especially for those related to GEOs, and the short period fluctuation is consistent with that of the MP series. After being smoothed with a Hatch filter, the fluctuation could not be cancelled even for a short baseline. As a result, DD wide-lane ambiguities that are related to GEOs cannot be fixed or it takes a very long time to converge, from one hour to several hours.

For example, the DD MW combination series of C01-C02 (GEOs) in Figure 12 was dominated by a low-frequency multipath and slowly fluctuated between −17.5 and −14.5 within 24 h, which is similar to the fluctuation of the MP series of C01 and C02, and the measurement noise was very small compared with that of IGSOs or MEOs only. The short period variations of DD MW combinations led to the failure of WL AR for a few hours, sometimes for a whole day, because wild-lane ambiguities may contain a half-cycle bias after long-time converging. The DD MW combination series between C01 and C10, C14 also demonstrate short period variations that need a long time to converge. For the DD MW combination series between C06 and C07, C10 and C14, and C12 and C14, a low-frequency multipath component could also be found, but its frequency was much higher than that of the DD MW combination series between C01 and C02, which did not affect WL AR.

That is to say, for Leica receivers, the DD MW combinations of GEOs of BDS contained an obvious low-frequency multipath, which seriously affects WL AR, even for short baseline. Although satellite-included bias existed for BDS, it did not affect WL AR for a short and medium baseline for Trimble and Septentrio receivers. This also suggests that the multipath may result from the Lecia receiver.

In order to confirm the above characteristics of BDS MW combinations for different receiver types, further analysis was conducted with different receiver types, as shown in Table 2. The fixing rate and the convergence time of WL AR are shown in Table 3 and Table 4 for different receiver types and different types of BDS satellites.

The integer rounding method [26] was employed to resolve the WL ambiguities in this paper. Firstly the true ambiguities were obtained by rounding the averaged float value of every continual data arcs of each pair of satellites [16]. Then, given a threshold of 0.25 [27,28], the WL ambiguity fixing rate is the result of dividing the number of fixed ambiguities by the number of ambiguities. 

As expected, the fixing rates of WL AR for Trimble and Septentrio receivers were very high for short and medium baselines. For example, in this experiment, the fixing rates of three types of satellites were over 97.6% for HKLM-HKQT. When baseline distance increased to 266.1 km, GEO-related fixing rates were comparable with those of a short baseline, while slightly lower for non-GEOs. With the increase of baseline distance, fixing rates become lower or the convergence time becomes longer, which may be a result of satellite-included elevation-dependent code bias. A similar conclusion could be obtained for Septentrio; for baseline NNOR-YAR2, the fixing rate and convergence time were comparable with Trimble’s at the same distance level.

For Leica receivers, meanwhile, although baseline distance was only dozens of kilometers, GEO-related fixing rates of WL AR were much lower, and the convergence time was much longer than these of Trimble (there were no short baseline data for Septentrio) at the same baseline distance level. Fixing rates and the convergence time of non-GEO-related satellites are comparable with the other two types of receivers listed in Table 3. As shown in Table 3 and Table 4, the baseline distances of HKMW-HKSL and HKMW-HKWS were 15.0 km, 39.5 km respectively, fixing rates and convergence time For GEO-related satellites, the fixing rates were much lower and the convergence time of some satellite pairs was very long. For example, the average convergence time of GEO-GEO for HKMW-HKSL was 251.0 epochs (epoch/30s), which means WL ambiguity cannot be fixed for about 2.1 h, and its fixing rate is only 73.3%. The fixing rates of GEO-IGSO and GEO-MEO were 78.1% and 72.4%, respectively, which was much lower than those of baselines with Trimble and Septentrio receiver. Convergence time was also much longer than that of Trimble and Septentrio receiver baselines. A similar phenomenon could also be found for HKMW-HKWS. The convergence time of GEOs for NNOR-YAR2 and MOBS-HOB2 was also slight longer than that with Trimble receivers, which might be the result of a longer baseline distance and satellite-included multipath.

### 3.4. Performance of the Time-Difference Method for WL AR 

**Figure 14:**
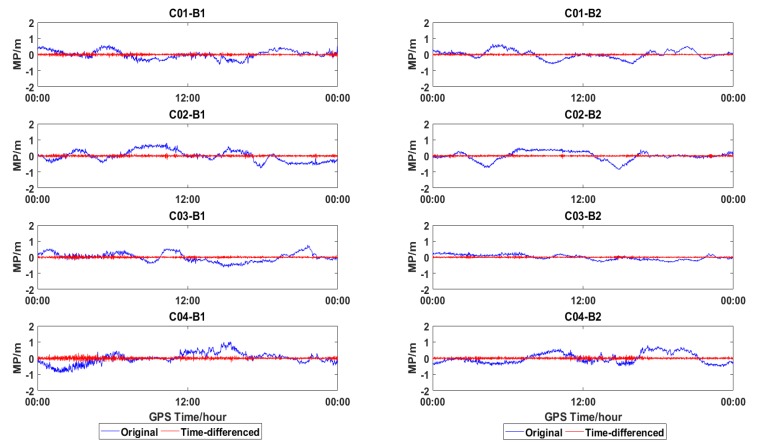
MP series of GEOs for HKMW (DOY 206).

Figure 14 shows the time-differenced series of MP, the residual only containing the measurement noise for multipath variation, which is very small between the adjacent epochs. The remaining measurement noise could easily be smoothed with Hatch filter.

**Figure 15:**
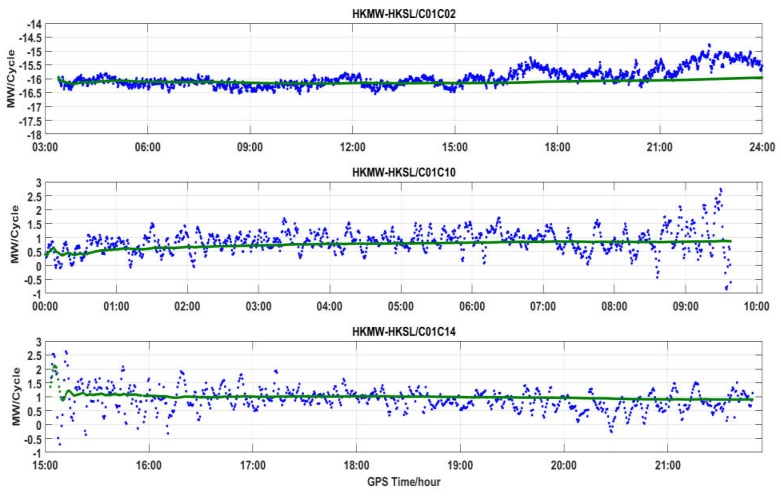
GEOs DD MW combinations of baseline HKMW-HKSL (Leica).

After correction with the proposed time-difference method, the DD WL ambiguities of C01-C02, C01-C10, and C01-C14 of HKMW-HKSL are shown in Figure 15, and the improvement is significant when comparing with Figure 12, both for fixing rate and convergence time, which means that the proposed method is effective for WL AR with a Leica receiver.

In order to further validate the proposed method, the same baselines with Lecia receivers in Table 2 were reprocessed with the time-difference correction method; fixing rate and convergence time are shown in Table 3 and Table 4 in bold font. For the baseline HKMW-HKSL, the fixing rates of GEO-GEO, GEO-IGSO, and GEO-MEO were improved to 94.1%, 95.20%, and 93.692.8% from 73.3%, 78.1%, and 72.4%, respectively, and the convergence time was shortened to 4.7, 8.9, and 8.4 epochs from 251.0, 45.0, and 40.0 epochs. Outstanding performance could also be found for HKMW-HKWS.

## 4. Discussions and Conclusions

Different types of receiver may adopt different internal multipath-mitigation methods and other techniques, which result in different characteristics of GNSS measurements; furthermore, it affects precise-positioning performance.

BDS data of different receiver types (including Trimble, Leica, Javad, and Septentrio), collected in the Asia-Pacific area, were processed and analyzed based on MP observables firstly. An obvious low-frequency component was found in BDS GEOs for the Leica receiver, that is, the obvious low-frequency component was the dominant of the MP observable for Leica receiver, and the dominant component was random measurement noise for the Trimble, Javad, and Septentrio receiver.

In order to confirm the existence of the low-frequency parts of the Leica receiver, daily repeatability and spatial similarity were analyzed with the correlation method and wavelet analysis. The result suggests a high similarity between the waveforms of GEOs MP series of the Leica receiver for two consecutive days; furthermore, for the receivers located in different places, a low-frequency component could also be observed. Then, the influence on WL AR was analyzed with four-week data during different seasons that showed that the low frequency significantly affects the WL AR, both for a fixed rate and convergence time as compared with the Trimble and Septentrio receiver. The fixed rate of the WL for a Leica receiver was only 59.2–78.1% for GEO-related satellites. Convergence time was also much longer than that of Trimble and Septentrio, as it needed several hours to converge for GEO-related satellites. 

Then, an improved WL AR method was proposed and validated. As the low-frequency component was the dominant part in the MP of BDS GEOs, and the Hatch filter could not work very well for WL AR, we could substitute the low frequency from the MW observation, and the WL could easily be resolved. The traditional method is employing the wavelet of FFT to retrieve low-frequency parts and substituting them on next sidereal day. The above analysis suggests that the raw MP series is dominated by low-frequency parts; we can directly substitute it from the MW observation. Considering the existence of high-frequency measurement noise, the time-difference method was adopted. The method was validated with four-week data. The result shows that the fixed rate was improved significantly and convergence time was also greatly shortened, both of which are comparable with those of Trimble and Septentrio with the proposed WL AR method.

For different receiver types, their internal multipath-mitigation methods are different, which leads to obvious different characteristics for MP and MW combination observables for BDS, and especially for GEOs. Although the antennas that were used in this study were different, it is expected that this difference could be neglected for all of them are geodetic-grade antennas.

Furthermore, the measurement characteristics from different receiver types are affected by many factors. In future research, we will confirm the low-frequency component with zero baseline data.

## Figures and Tables

**Table 1:** Header information of five Global Navigation Satellite System (GNSS) stations.

Site ID	Site Name	Receiver Type	Firmware Version	Antenna Type	Country
HKQT	Quarry Bay	TRIMBLE NETR9	5.22	TRM59900.00	China
HKLM	Hung Shing Yeh Beach	TRIMBLE NETR9	5.22	TRM59900.00	China
HKMW	Mui Wo	LEICA GR50	4.02.386/7.002	LEIAR25.R4	China
HKSL	Siu Lang Shui	LEICA GR50	4.02.386/7.002	LEIAR25.R4	China
HKWS	Wong Shek	LEICA GR50	4.02.386/7.002	LEIAR25.R4	China
LHAZ	Lhasa/Tibet/China	LEICA GR25	4.11/6.523	LEIAR25.R4 LEIT	China
CIBG	Cibinong	LEICA GR10	4.11/6.523	LEIAR25.R4 NONE	Indonesia
ALIC	Alice Springs AU012	LEICA GR25	4.11.606/6.523	LEIAR25.R3 NONE	Australia
PARK	Australian Telescope National Facility	TRIMBLE NETR9	5.22	ASH701945C_M NONE	Australia
STR2	Stromlo	TRIMBLE NETR9	5.22	LEIAR25.R3 NONE	Australia
YAR2	Yarragadee	SEPT POLARX4TR	2.9.6	AOAD/M_T NONE	Australia
NNOR	New Norcia	SEPT POLARX4	2.9.5-extref1	SEPCHOKE_MC NONE	Australia
MOBS	Melbourne Observatory	SEPT POLARX4TR	2.9.6	ASH701945C_M NONE	Australia
HOB2	Hobart AU016	SEPT POLARX5	5.10	AOAD/M_T NONE	Australia
GAMG	Geochang	SEPT POLARX4TR	2.9.6	LEIAR25.R4	Korea
MIZU	Mizusawa	JAVAD TRE_G3TH	3.6.7	JAV_RINGANT_G3T	Japan

**Table 2:** Baseline information.

Trimble		Leica		Septentrio	
Baseline	Distance	Baseline	Distance	Baseline	Distance
HKLM-HKQT	12.8	HKMW-HKSL	15.0	NNOR-YAR2	236.5
STR2-PARK	266.1	HKMW-HKWS	39.5	MOBS-HOB2	590.5

**Table 3:** Fixing rate of wide-lane ambiguity resolution (WL AR) (%).

	Baseline	Types of Satellites Pairs					
		GEO-GEO	GEO-IGSO	GEO-MEO	IGSO-IGSO	IGSO-MEO	MEO-MEO
Trimble	HKLM-HKQT	97.9	99.5	98.2	99.2	98.4	97.6
	STR2-PARK	93.6	92.7	89.6	89.4	87.8	89.4
Leica	HKMW-HKSL	73.3/**94.1**	78.1/**95.2**	72.4/**93.6**	92.22	91.0	90.9
	HKMW-HKWS	70.0/**94.6**	72.3/**91.5**	59.2/**87.7**	91.1	92.3	92.2
Septentrio	NNOR-YAR2	94.3	96.3	92.3	93.8	94.3	92.7
	MOBS-HOB2	86.5	91.1	87.6	97.7	95.6	93.5

**Table 4:** Convergence time of WL AR (Epoch/30 s).

	Baseline	Types of Satellites Pairs					
		GEO-GEO	GEO-IGSO	GEO-MEO	IGSO-IGSO	IGSO-MEO	MEO-MEO
Trimble	HKLM-HKQT	11.0	3.0	5.4	2.0	4.0	4.4
	STR2-PARK	15.6	3.4	3.7	4.6	13.0	9.3
Leica	HKMW-HKSL	251.0/**4.7**	45.0/**8.9**	40.0/**8.4**	5.5	7.5	7.4
	HKMW-HKWS	354.0/**1.8**	58.3/**7.7**	34.7/**19.5**	5.8	7.1	5.8
Septentrio	NNOR-YAR2	56.7	5.7	8.2	1.9	2.3	2.1
	MOBS-HOB2	40.7	12.0	8.7	2.0	3.6	5.3

Bold: fixing rate and convergence time with the time-difference correction method.
